# Application of weighted gene co-expression network and immune infiltration for explorations of key genes in the brain of elderly COVID-19 patients

**DOI:** 10.3389/fimmu.2023.1157179

**Published:** 2023-03-31

**Authors:** Lixia Huang, Wei Qin, Zirui Guo, Xiaoyu Li, Fajiu Li, Xiang Wang

**Affiliations:** ^1^ Department of Immunology, School of Medicine, Jianghan University, Wuhan, China; ^2^ Tianyuan Translational Medicine R&D Team, School of Medicine, Jianghan University, Wuhan, China; ^3^ Department of Pulmonary and Critical Care Medicine, Affiliated Hospital of Jianghan University, Wuhan, China; ^4^ Institution of Pulmonary Vascular Disease, Jianghan University, Wuhan, China; ^5^ Department of Materials (D-MATL), ETH Zurich, Zurich, Switzerland; ^6^ Department of Histology and Embryology, School of Medicine, Jianghan University, Wuhan, China

**Keywords:** COVID-19, lung-brain axis, long COVID, brain damage, sequelae, TIMP 1, RPS 29, S100A10

## Abstract

**Introduction:**

Although many studies have demonstrated the existing neurological symptoms in COVID-19 patients, the mechanisms are not clear until now. This study aimed to figure out the critical molecular and immune infiltration situations in the brain of elderly COVID-19 patients.

**Methods:**

GSE188847 was used for the differential analysis, WGCNA, and immune infiltration analysis. We also performed GO, KEGG, GSEA, and GSVA for the enrich analysis.

**Results:**

266 DEGs, obtained from the brain samples of COVID-19 and non-COVID-19 patients whose ages were over 70 years old, were identified. GO and KEGG analysis revealed the enrichment in synapse and neuroactive ligand-receptor interaction in COVID-19 patients. Further analysis found that asthma and immune system signal pathways were significant changes based on GSEA and GSVA. Immune infiltration analysis demonstrated the imbalance of CD8+ T cells, neutrophils, and HLA. The MEpurple module genes were the most significantly different relative to COVID-19. Finally, RPS29, S100A10, and TIMP1 were the critical genes attributed to the progress of brain damage.

**Conclusion:**

RPS29, S100A10, and TIMP1 were the critical genes in the brain pathology of COVID-19 in elderly patients. Our research has revealed a new mechanism and a potential therapeutic target.

## Introduction

Corona Virus Disease 2019 (COVID-19) is one of the biggest public health challenges in the world. It is a highly contagious disease. According to the latest data from the World Health Organization (WHO), as of January 17, 2023, there have been more than 660 million cases of COVID-19 worldwide, with approximately 6.69 million cumulative deaths (1). Once infected with Severe Acute Respiratory Syndrome Coronavirus 2 (SARS-CoV-2), the excessive inflammatory response can lead to lung injury, including microvascular and endothelial dysfunction, and subsequent coagulation disorders and thrombosis, especially in severe and critically ill patients. Although COVID-19 is a respiratory disease, there is sufficient evidence that SARS-CoV-2 also causes multi-organ dysfunction throughout the body, including the heart, kidneys, digestive system, and nervous system (2, 3). Meanwhile, some patients continue to experience long symptoms after recovery, which is termed “long COVID” (4–6).

Regardless of the severity of the disease, COVID-19 can present a myriad of neurological signs and symptoms throughout its course (7, 8). These symptoms can occur in the acute and/or recovery phase of the infection. Among the various neurological symptoms, the most prevalent is loss of taste and smell, which has been reported in up to 53% and 44% of patients in one study, respectively (9). In addition, it was reported that severe COVID-19 patients that are young (≤38 years old) have strikingly similar genetic regulation with much older (≥71 years old) uninfected individuals (8) Similarly, other symptoms were also reported, such as cognitive impairment, seizures, and cerebrovascular disease.

Given the various neurological manifestations of COVID-19, the mechanisms of neurologic injury may be multifactorial. After carrying out complete autopsies on 44 patients who died of COVID-19, Sydney R. Stein et al. revealed that SARS-CoV-2 can spread across the whole body, predominantly in the case of severe COVID-19 patients, and that virus replication is present in multiple respiratory and non-respiratory tissues, including the brain (10). Notably, despite the extensive distribution of SARS-CoV-2 RNA throughout the body, they observed little evidence of inflammation or direct viral cytopathology outside the respiratory tract. This can explain the long time it takes for the virus to be cleared in the body and the systemic distribution of the virus preliminarily, but it is not sufficient to elucidate the mechanism of many neurological-related symptoms brought by SARS-CoV-2.

In this study, we combined differentially expressed analysis, weighted gene co-expression network analysis (WGCNA), enrichment analysis, immune infiltration analysis, and key molecule identification, to identify the critical genes causing neurological symptoms in elderly COVID-19 patients and the possible pathogenesis.

## Materials and methods

### Data acquisition and differential gene analysis

GSE188847 was downloaded from gene expression omnibus (GEO, https://www.ncbi.nlm.nih.gov/geo/). We collected human brain tissue samples from COVID-19 patients and uninfected patients aged >70 years based on the previous study, with a total of 7 control and 6 COVID-19 patient samples (8). We used |logFC|>1 and *p*-value< 0.05 as filtering conditions and used the limma package normalize Between Arrays function for data correction, resulting in 266 differential genes (DEGs).

### Functional and pathway enrichment analysis

For differential genes, The Gene Ontology (GO) and Kyoto Encyclopedia of Genes and Genomes (KEGG) enrichment analysis of differential genes was performed using the cluster Profiler package with q-value < 0.05 as the statistically significant criterion. To reduce bias, we used GSEA (Gene Set Enrichment Analysis) and GSVA (Gene set variation analysis) (http://software.broadinstitute.org/gsea/index.jsp) for enrichment analysis because GO and KEGG were only applicable for differential genes. In GSEA analysis, we chose c2.cp.kegg.v7.4 as the annotated gene set with *p* < 0.05 and gene number > 15 as the filtering condition, where NES > 0 is enriched in COVID-19 and NES < 0 is enriched in con. In GSVA analysis, we selected c2.cp.kegg.v7.2 as the annotated gene set with *p* < 0.05 and gene number >10 as the filtering condition.

### Evaluation of immune cell distribution and immune function in the brain of COVID-19 patients

In the GSE188847 dataset, we assessed the infiltration of 22 immune cell types in the brain of COVID-19 patients aged >70 years. The “CIBERSORT.R” code and the standard immune cell expression file are available from the official website (https://cibersort.stanford.edu/). The data were then analyzed in the R software using the limma package. Based on the expression of RPS29, the patients were divided into two groups with high and low expression; based on the immune cell infiltration of the patients, immune function assessments of the high and low expression groups were performed.

### WGCNA analysis

The genes with 0 expressions in GSE188847 were deleted and all remaining genes were used for WGCNA analysis. A hierarchical clustering analysis is first performed, and we select 90 as the sample inclusion criterion to eliminate outliers in the sample. Then soft thresholds were calculated and optimal values were selected for subsequent network construction to obtain the real biological network state (scale-free network). When the scale-free index (R2) reached 0.90 and the average connectivity was close to 0, 7 is chosen as our soft threshold. The weighted gene co-expression network was constructed by the optimal soft threshold power based on gene expression correlation, and the co-expression modules were identified and clustered based on their similarity to each other. The minimum number of genes in each clustered module was set to 100 after determining the relationship and correlation between these clustered modules and phenotypes, the gene significance (GS) and module affiliation (MM) between each module and COVID-19 were calculated and visualized. Finally, the module with the highest correlation coefficient was selected as the key module and applied further analysis.

### Aggregation analysis and identification of key molecules in the purple module

For the aggregation analysis of purple modules, GO and KEGG enrichment analysis of module genes were performed using the cluster Profiler package with q-value < 0.05 as the judgment criterion. Using COVID-related genes (https://www.kegg.jp/), differential and modular genes were taken to intersect to obtain RPS29, and by correlation analysis, intersected with DEGs to obtain TIMP1. The same method was used for immune genes (https://www.innatedb.com/ and https://immport.org/shared/home), differential, and modular genes to obtain S100A10, and by correlation of expression, intersected with differential genes to obtain an intersection set to obtain TIMP1. Finally, the scatter plot of TIMP1 was plotted to observe its expression. The COVID patients were divided into high and low-expression groups according to the expression of TIMP1, and GSEA enrichment analysis was performed for the high and low-expression groups.

## Results

### Identification of DEGs and enrichment analysis in the brain of COVID-19 patients

**Figure 1:**
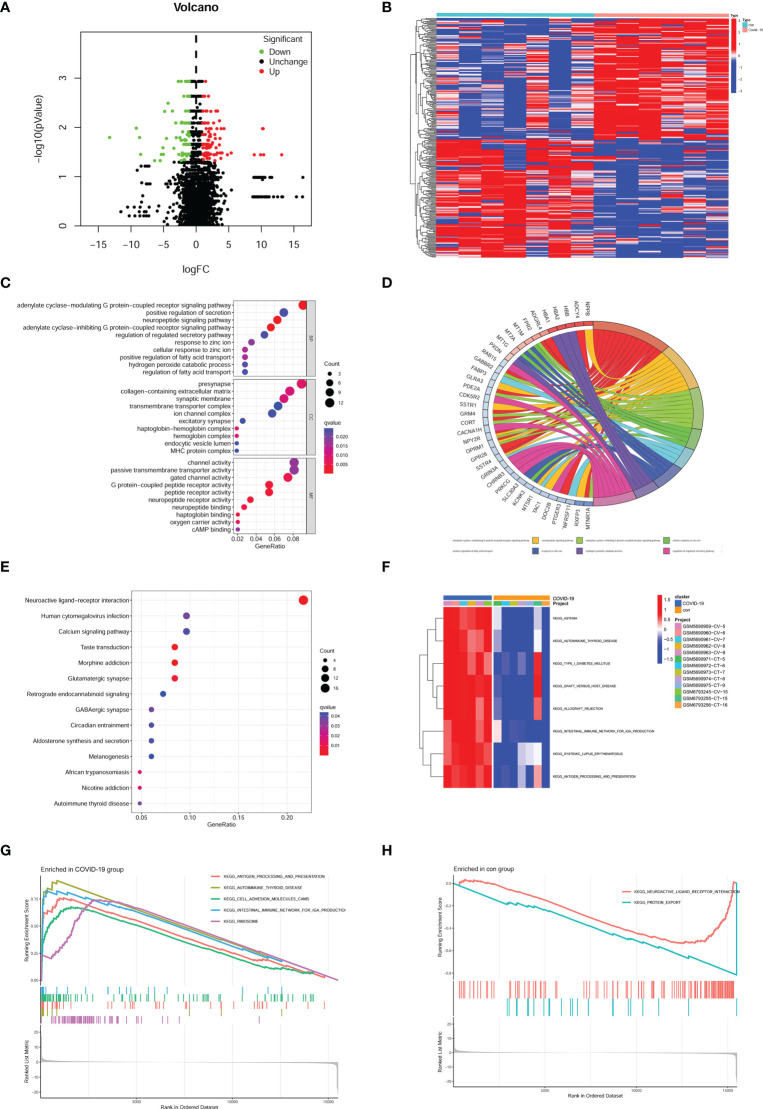
Identification of DEGs and enrichment analysis in the brain of COVID-19 patients. **(A)** Volcano plot of DEGs in GSE188847; Red plots, up-regulated genes; Green plots, down-regulated genes; Black plots, unchanged genes. **(B)** Heatmap of DEGs. **(C)** bubble plot of GO analysis. **(D)** According to the |logFC| the most relative genes were displayed. **(E)** bubble plot of KEGG analysis. **(F)** the result of GSVA analysis. **(G)** the result of GSEA analysis enriched in the COVID-19 group, **(H)** the result of GSEA analysis enriched in the con group.

266 DEGs were identified from two groups (healthy and COVID-19 patients) based on *p* value < 0.05 and |logFC| > 1. Up-regulated and down-regulated genes were presented as red and green plots in the volcano plot (**Figure 1A**). The gene expression of DEGs was shown in a heatmap plot (**Figure 1B**). To gain further insight into their potential functions, GO and KEGG analyses were performed on DEGs. GO terms include biological process (BP), cellular component (CC), and molecular function (MF). Our results showed 36 BP terms (including adenylate cyclase-modulating G protein-coupled receptor signaling pathway, neuropeptide signaling pathway, adenylate cyclase-inhibiting G protein-coupled receptor signaling pathway, cellular response to zinc ion), 13 CC terms (presynapse, haptoglobin-hemoglobin complex, hemoglobin complex, collagen-containing extracellular matrix and more), 44 MF terms (G protein-coupled peptide receptor activity, neuropeptide receptor activity, peptide receptor activity, haptoglobin binding and more) (**Figure 1C**). According to their |logFC|, genes were shown the relationship with the first eight BP terms (**Figure 1D**). KEGG results suggested that several signal pathways were aberrantly changed in COVID-19 patients’ brains, such as Neuroactive ligand-receptor interaction, Taste transduction, Morphine addiction, and Glutamatergic synapse (**Figure 1E**). GSEA and GSVA analyses were also performed to prevent bias. GSVA analysis suggested several signal pathways were significantly enriched in the COVID-19 group, such as asthma, graft versus host reaction, allograft rejection, autoimmune thyroid disease, and intestinal immune network (**Figure 1F**). Meanwhile, GSEA analysis suggested that ribosome, autoimmune thyroid disease, antigen processing, presentation, and cell adhesion molecules were enriched in the COVID-19 group (**Figure 1G**), while neuroactive ligand-receptor interaction and protein export were enriched in the control group (**Figure 1H**).

### Immune landscape in the brain of COVID-19 patients

**Figure 2:**
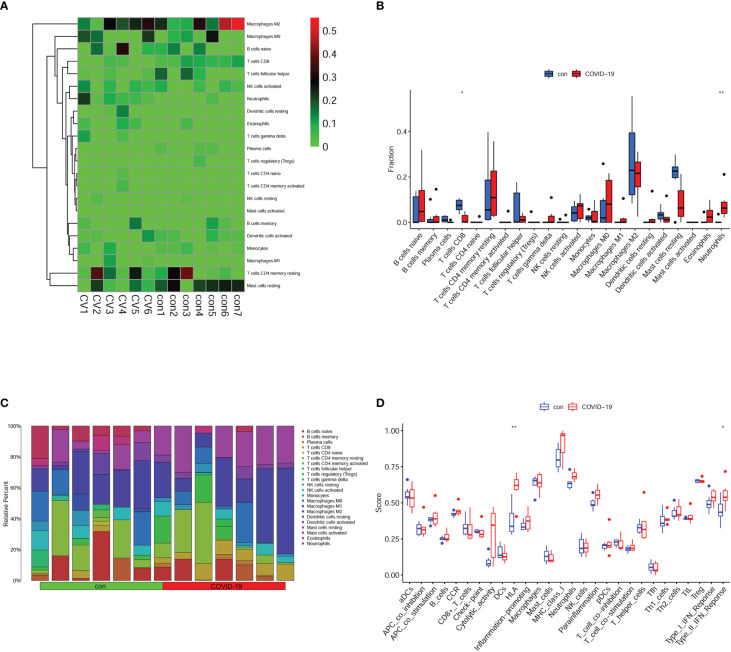
Immune landscape in the brain of Covid-19 patients. **(A)** immune cell distribution in each sample. **(B)** immune cell fraction in the brain of control and Covid-19 patients. **(C)** relative percent of immune cells in each sample. **(D)** immune function in the brain of control and Covid-19 patients. *p < 0.05; **p < 0.01.

Our enrichment analysis suggested that immune-related signal pathways were enriched in the brain of COVID-19 patients. Following that, we conducted immune infiltration and immune function analysis in GSE188847. The distribution of different kinds of immune cells was shown in each sample (**Figure 2A**). The immune landscape and proportion results revealed that CD8+ T cells were downregulated and Neutrophils were upregulated in COVID-19 patients (**Figures 2B, C**). Meanwhile, immune function analysis uncovered that HLA was significantly upregulated (**Figure 2D**). Our immune situation analysis results indicated that immune system dysfunctions occurred in the brain of COVID-19 patients.

### Construction of weighted gene network and key module identification

**Figure 3:**
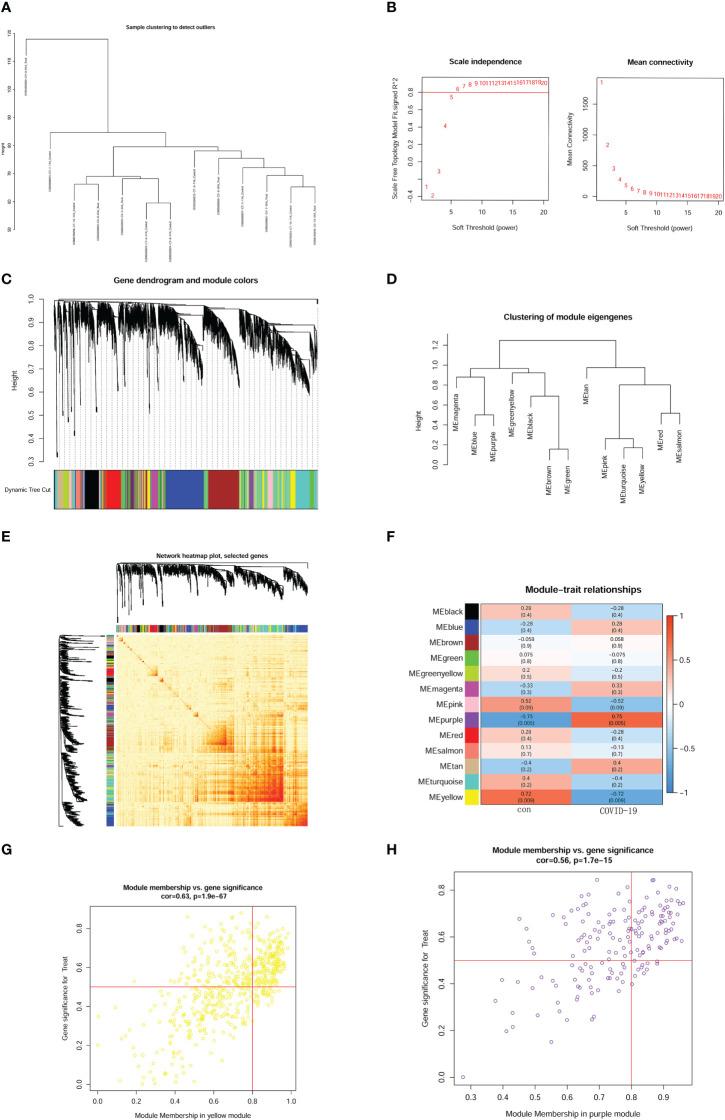
Construction of weighted gene network and key module identification. **(A)** sample clustering. **(B)** threshold power confirmed. The left panel shows the scale-free fit index. The right panel represented mean connectivity. **(C)** gene dendrogram according to the value of dissimilarity measure (1-TOM). **(D)** enrich analysis of module eigengenes. **(E)** heatmap of genes based on distance on each other. **(F)** module-trait relationships and definite relativity and p-value. **(G)** module membership (MM) vs. gene significance analysis in MEyellow module. **(H)** module membership (MM) vs. gene significance analysis in MEpurple module.

Heterogeneity detection was conducted for each sample through hierarchical clustering analysis to check and remove outliers (**Figure 3A**). All genes from the 12 samples were performed WGCNA (weighted correlation network analysis) analysis. The threshold power was 7 when R2 reached 0.9 and the average connectivity was near 0 (**Figure 3B**). Therefore, we used the power value 7 to perform WGCNA network construction. To evaluate the relationship and generate a module of closely related genes, we performed a hierarchical clustering tree analysis, as shown in **Figure 3C**. We also investigated the correlation between different modules (**Figure 3D**). According to the distance of different genes between each other, we created a heatmap (**Figure 3E**). WGCNA network module-trait relationships were conducted to observe the most correlated modules. In the analysis, we found that MEpurple (*p* = 0.005) and MEyellow (*p* = 0.009) had a significant correlation with the brain prognoses in COVID-19 patients, which suggested that the genes in those modules have a positive or negative effect on the development of brain pathology in COVID-19 patients. Remarkably, MEpurple had the strongest correlation (r = 0.75) and the lowest *p*-value (*p* = 0.005) (**Figure 3F**). After identifying the key module, we conducted module membership (MM) vs. gene significance analysis in MEyellow (**Figure 3G**) and MEpurple (**Figure 3H**) modules. Finally, the MEpurple module was identified as the critical module for the brain progression of COVID-19 patients.

### Enrichment analysis on MEyellow module genes

**Figure 4:**
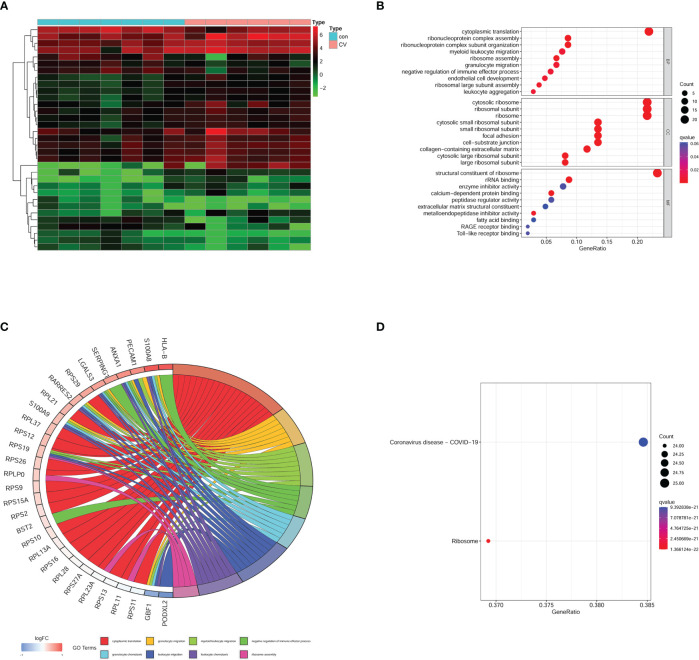
Enrichment analysis on MEpurple module genes. **(A)** heatmap of gene expression in MEpurple module. **(B)** GO analysis. **(C)** According to the |logFC| the most relative genes was displayed. **(D)** KEGG analysis. Con, control group; CV, COVID-19 group.

We used a heatmap to display the expression of MEpurple module genes (**Figure 4A**). All genes in MEpurple module were also conducted GO and KEGG analyses. In GO analysis BB terms implied that several biological processes were disrupted, such as cytoplasmic translation, ribonucleoprotein complex assembly, myeloid leukocyte migration, and ribosome assembly. CC terms were enriched in the cytosolic ribosome, ribosomal subunit, cytosolic small ribosomal subunit, and the ribosome. MF term also suggested the most significant impact happened to the structural constituent of ribosome (**Figure 4B**). The results implied ribosome damage on the functionality and component., The correlation genes with the first eight BP terms were conserved in the chord diagram according to |logFC| (**Figure 4C**). KEGG analysis uncovered synaptic dysfunction signal pathways as shown in **Figure 1E**. Interestingly, the most enriched pathway was COVID-19 in MEpurple module genes (**Figure 4D**). Our results provided strong evidence that COVID-19 could not only cause multiple organ dysfunction but also brain damage.

### Critical genes in enriched pathways

**Figure 5:**
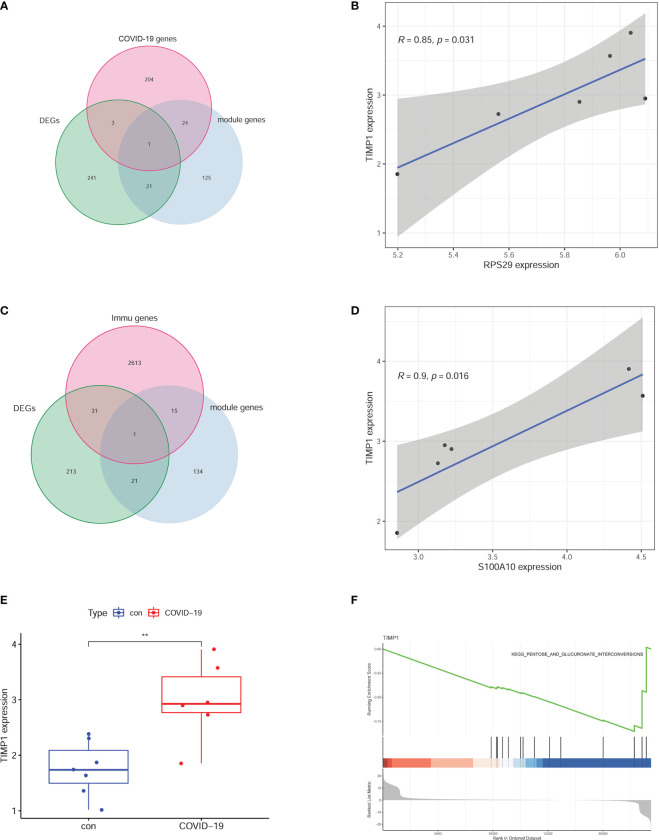
Critical molecules based on the signal pathway. **(A)** RPS29 was selected by intersecting the DEGs, MEpurple module genes, and COVID-19 genes. **(B)** the correlative analysis between RPS29 and TIMP1. **(C)** S100A10 was chosen by intersecting the DEGs, MEpurple module genes, and immune genes. **(D)** the correlative analysis between S100A10 and TIMP1. **(E)** different expressions of TIMP1. **(F)** GSEA analysis according to TIMP1 expression. **p < 0.01.

Because of the inflammatory storm caused by COVID-19 in several organs, we explored the critical immune molecule in the brain of COVID-19 patients. The COVID-19-related genes, DEGs, and module genes were selected to obtain the intersection gene. As a result, RPS29 was identified (**Figure 5A**). The correlation between RPS29 and other gene expressions was analyzed by Pearson using a corrplot package according to correlation > 0.7 and *p*-value < 0.05. The TIMP1 was the most relevant with RPS29 in DEGs as shown in **Figure 5B**. For the critical role of the immune pathway in the brain of COVID-19 patients, the intersection genes were obtained by intersecting immune pathway genes, DEGs, and MEpurple genes (**Figure 5C**). The correlated genes with S100A10 were analyzed by Pearson using corrplot package and significant genes were shown in the cyclograph according to correlation > 0.7 and *p*-value < 0.05. TIMP1 was the most related gene with S100A10 in DEGs (**Figure 5D**). To further explore the expression and role of TIMP1, we found that the expression of TIMP1 was significantly elevated in the brains of COVID-19 patients by scatter plots (**Figure 5E**). Based on the expression of TIMP1, further analysis by GSEA revealed that the pentose and glucuronate interconversions pathways were significantly enriched in the TIMP1 low expression group, indicating that the metabolic pathways in the brain of COVID-19 patients were disrupted (**Figure 5F**).

### Immune landscape based on RPS29 expression in COVID-19 patients

**Figure 6:**
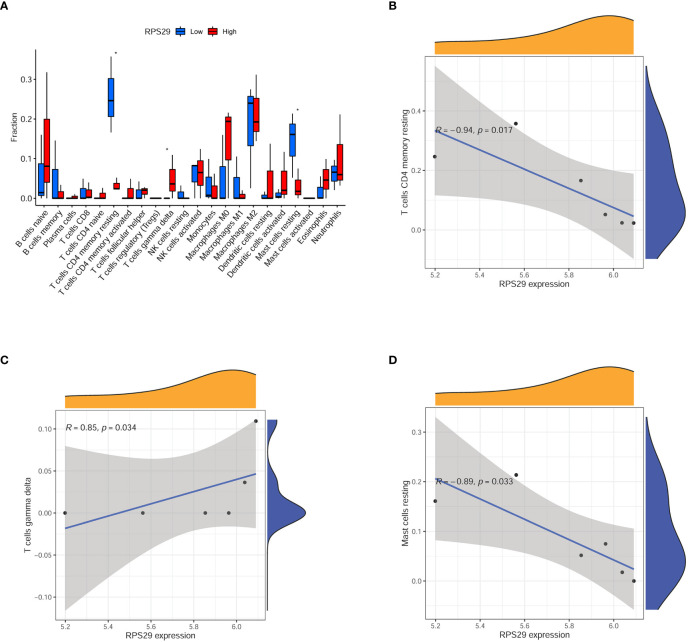
Immune infiltration analysis according to RPS29 expression in the brain of Covid-19 patients. **(A)** immune cell fraction between low and high expression of RPS29 in the brain of Covid-19 patients. **(B)** the correlation between RPS29 expression and T cells CD4 memory resting. **(C)** the correlation between RPS29 expression and T cells gamma delta. **(D)** the correlation between RPS29 expression and mast cells resting. *p < 0.05.

To explore the relationship between immune situation and RPS29 expression, we divided the COVID-19 patients into two groups (low expression group and high expression group) according to the median value of RPS29 expression. It was found that T cells CD4 memory resting, T cells gamma delta, and Mast cells resting were significantly different in the two groups (**Figure 6A**). Among them, the most relevant was immune cell T cells CD4 memory resting (**Figure 6B**). The correlation of RPS29 expression with T cells gamma delta and Mast cells resting was also shown in **Figures 6C, D**.

## Discussion

Patients infected by SARS-CoV-2 may develop a wide range of clinical manifestations including not only pneumonia, acute respiratory distress syndrome (ARDS), and respiratory failure but also systemic inflammation and multiorgan failure as well as a range of chronic residual symptoms (2, 11, 12). Among the more prominent are neurological symptoms, including encephalopathy and/or cerebrovascular diseases. Some discharged patients have reported collective symptoms of “brain fog”, i.e. impairments in attention, executive function, language, reaction speed, and memory (13, 14). Unfortunately, the available evidence does not yet fully explain the exact mechanism by which these clinical symptoms occur in the acute and recovery phases (5, 14).

In the current study, a total of 266 DEGs were identified. GO and KEGG analysis were enriched in synapse and neuroactive ligand-receptor interaction, this indicated the possible mechanisms of neurological pathologies. Further analysis based on GSEA and GSVA found that asthma and immune system genes were significantly affected. Intracranial genes were co-expressed with intrapulmonary genes indicating the potential correlation along the lung-brain axis. The past report demonstrated lung infections and smoking as risk factors for multiple sclerosis, a T-cell-mediated autoimmune disease of the central nervous system (15). After cigarette smoke exposure in the COPD model, it was observed that systemic inflammation is associated with increased exploratory behavior, suggesting that neuroinflammation was present in the brain area involved in cognitive functioning and that blood-brain barrier integrity was compromised (16). Immune infiltration analysis demonstrated the imbalance of CD8+ T cells, neutrophils, and HLA, indicating the dysregulation of intracranial immune function. CD8+ T cells can kill infected cells and the presence of virus-specific CD8+ T cells was found to be associated with a positive prognosis of COVID-19 in SARS-CoV-2 infection (17). However, the CD8+ T cells gene in the brain had an expression decrease in patients who died of COVID-19. The HLA system coordinates immune regulation and plays a critical role in the immune response to pathogens and the development of infectious diseases. The interaction between HLA and viruses is complex, so there is room for further research on the role of HLA in COVID-19 (18). Innate immune cells, including macrophages, monocytes, dendritic cells, neutrophils, and innate lymphoid cells (ILCs) such as natural killer (NK) cells, have a repertoire of pattern recognition receptors (PRRs) that recognize pathogen-associated molecular patterns (PAMPs) or damp-associated molecular patterns (DAMPs) to induce inflammatory signaling pathways and immune responses (19). Our results also showed that innate immune cells, i.e. neutrophils, were upregulated in brain tissue, playing an important role in brain inflammation.

Previous reports have shown that there are 16 non-structural proteins (NSP1–NSP16) that encode the RNA-directed RNA polymerase, helicase, and other components required for virus replication (20). One of the reported roles of NSP1 in SARS-CoV-2 is that it can associate with the 40S ribosome to inhibit host mRNA translation (21, 22). NSP1 binds to 18S ribosomal RNA in the mRNA entry channel of the ribosome and leads to global inhibition of mRNA translation upon infection (23). GO and KEGG analysis of the purple module genes showed significant enrichment of ribosome-related gene expression and function, suggesting that ribosomal dysfunction plays a key role in brain dysfunction in elderly COVID-19 patients. Pathway analysis suggested upregulation of 40S Ribosomal Protein S29 (RPS29). Therefore, we speculate that SARS-Cov-2 probably affects brain function through the regulation of RPS29.

S100A10 is a member of the S100 family of proteins containing 2 EF-hand calcium-binding motifs. Previous studies have shown that the mRNA expression of S100A4 (FC = 1.43, *p* = 0.0071), S100A9(FC = 1.66, *p* = 0.0001), and S100A10 (FC = 1.63, *p* = 0.0003) were significantly upregulated in the severe COVID-19 subjects than mild-to-moderate subjects based on 65 COVID-19 subjects and 50 healthy controls (24) These sound evidence indicated that S100A4, S100A9, and S100A10 play a role in the inflammatory conditions in COVID-19 patients and lead the onset of serious illness. A positive correlation of S100A8/A9 may serve as a predictive biomarker for thromboembolism and tissue injury in COVID-19 (25). Likewise, our results showed that the expression of S100A10 was significantly elevated which supported the possibility of inflammation in the brain.

Stefano Brusa et al. pointed out that in patients with COVID-19, circulating TIMP-1 was associated with disease severity and systemic inflammatory index (26). Upregulation of TIMP-1 expression has been reported to be associated with liver and lung fibrosis (27–29). Meanwhile, TIMP-1 is a potential element in the development of the asthmatic phenotype (30, 31). Recent studies have shown that elevated levels of MMP regulator TIMP-1 are an indication of a TIMP-1-mediated host antiviral response in the brain (32). In this study, we found a marked increase in TIMP1 expression in the brain of COVID-19 patients. Interestingly, TIMP-1 is the most related differential gene between RPS29 and S100A10, these results demonstrate the presence of intracranial inflammatory response in COVID-19 patients. Further results of GSEA analysis of TIMP-1 indicated abnormalities in the pentose and glucuronate interconversion signaling pathway, which is strongly associated with diabetes (33). Interestingly, the mortality rate of SARS-Cov-2 infection combined with diabetes was significantly higher than that of COVID-19 patients without diabetes (34), further illustrating the key role of TIMP-1 in COVID-19 pathological development.

Overall, our results showed that the total immune infiltration in the brain of elderly patients with SARS-Cov-2 infection showed abnormalities in both innate and adaptive immunity, especially in neutrophil infiltration. Also, our pathway enrichment analysis showed abnormal brain function, especially synaptic dysfunction, suggesting the involvement of the immune system in brain dysfunction. Further WGCNA analysis suggested that ribosomal dysfunction may play a key role in brain dysfunction in elderly COVID-19 patients, with RPS29, S100A10, and TIMP1 as key molecules. In conclusion, our results provided the first systematic analysis of the immune profile and the mechanisms of symptom occurrence in brain tissues in elderly COVID-19 patients. More importantly, we identified key molecules and provided new ideas for encephalopathy in elderly COVID-19 patients.

In the present study, although we were able to identify key molecules with statistical significance, some limitations need to be elucidated. Firstly, because of the rapid mutation of SARS-Cov-2, the results of this study are not representative of the pathological features caused by all types of virulent strains. Secondly, the sample size in this study was relatively small and the analysis was based on a single sequencing dataset, which was not validated. Lastly, our study was based on a population older than 70 years old in accordance with the previous study. Therefore, the results apply only to that group of individuals.

## Conclusion

COVID-19 patients may suffer from brain damage. Altered synaptic function and ribosome function may contribute to neurological pathological manifestations. RPS29, TIMP-1, and S100A10 may be the key molecules in craniofacial damage and immune function impairment.

## Data availability statement

The original contributions presented in the study are included in the article/supplementary material. Further inquiries can be directed to the corresponding authors.

## Ethics statement

Ethical review and approval was not required for the study on human participants in accordance with the local legislation and institutional requirements. Written informed consent for participation was not required for this study in accordance with the national legislation and the institutional requirements.

## Author contributions

XW, WQ, FL, and LH conceived the study. XW and XL collected data. XW, WQ, and ZG analyzed and interpreted data. XW and WQ drafted the manuscript. ZG, FL, and LH revised the manuscript. All authors contributed to the article and approved the submitted version.
